# Tight junctions and their regulation by non-coding RNAs

**DOI:** 10.7150/ijbs.45885

**Published:** 2021-01-31

**Authors:** Xiaojiao Zhao, Hongliang Zeng, Li Lei, Xiaoliang Tong, Lun Yang, Yan Yang, Si Li, Ying Zhou, Liping Luo, Jinhua Huang, Rong Xiao, Jing Chen, Qinghai Zeng

**Affiliations:** 1 Department of Dermatology, Third Xiangya Hospital, Central South University, 138 Tongzipo Road, Changsha, Hunan 410013, P.R. China.; 2 Institute of Chinese Materia Medica, Hunan Academy of Chinese Medicine, Yuehua Road, Changsha, Hunan 410013, P.R. China.; 3 Department of Dermatology, Second Xiangya Hospital, Central South University, 139 Renminzhong Road, Changsha, Hunan 410013, P.R. China.

**Keywords:** tight junction, micro-RNAs, long-noncoding RNAs, circular RNAs

## Abstract

Tight junction (TJ) is a “zippering up” junction structure located at the uppermost portion of adjacent epithelial/endothelial cells in organs and tissues. TJs maintain the relative stability of intracellular substances and functions by closing or opening intercellular pathways, coordinating the entry and exit of molecules of different sizes and charges, and regulating the permeability of paracellular barrier. TJs also prevent microbial invasion, maintain epithelial/endothelial cell polarity, and regulate cell proliferation. TJs are widely present in the skin and mucosal epithelial barriers, intestinal epithelial barrier, glomerular filtration barrier, bladder epithelial barrier, blood-brain barrier, brain-blood tumor barrier, and blood-testis barrier. TJ dysfunction in different organs can lead to a variety of diseases. In addition to signal pathways, transcription factors, DNA methylation, histone modification, TJ proteins can also be regulated by a variety of non-coding RNAs, such as micro-RNAs, long-noncoding RNAs, and circular RNAs, directly or indirectly. This review summarizes the structure of TJs and introduces the functions and regulatory mechanisms of TJs in different organs and tissues. The roles and mechanisms of non-coding RNAs in the regulation of TJs are also highlighted in this review.

## Introduction

Mammals have four types of cell-cell junction that called tight junctions (TJs), adherens junctions (AJs), gap junctions (GJs), and desmosomes/hemidesmosomes 1. According to functions, they can be divided into occluding junctions (TJs), anchoring junctions (AJs, desmosome/hemidesmosome), and communicating junctions (GJs) 2-4. These junctions jointly regulate the paracellular or intra-/extracellular exchanges of material and information 5. Among them, TJs are paracellular barriers that are essential for maintaining homeostasis in multicellular organisms and can quickly respond to stimuli, especially in the skin, visceral organs, and blood-brain barrier (BBB) 6. As selective gates, TJs control the paracellular diffusion of ions and solutes, allow the passage of small soluble substances, restrict the exudation of macromolecular substances, such as proteins, and inhibit the entry of viruses and other microorganisms 5, 7, 8.

## TJ structure

**Figure 1:**
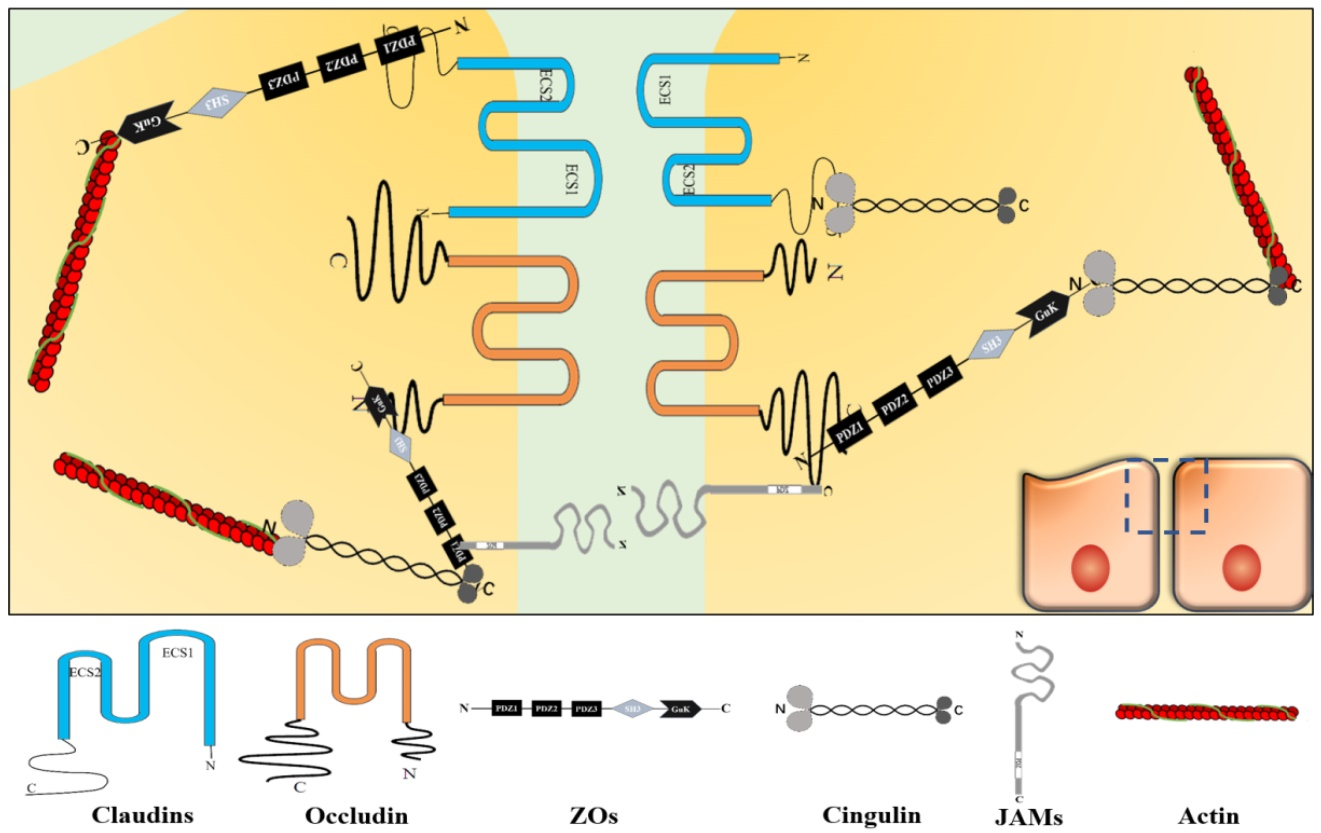
**TJ structure**. Claudins, occludin, JAM, and etc. constitute the core components of TJ strand. Among them, claudins aggregate to form the backbone of the TJ strand. ZO protein family, cingulin, and etc. act as scaffold proteins to connect TJ strand and actin in TJ. TJ shows a reticular structure formed by the orderly binding of different functional proteins.

TJs are located in the topmost region of the lateral membrane 9. A TJ is a reticular structure formed by the binding of transmembrane proteins (claudins, occludin, junctional adhesion molecule [JAM], etc.), cytoplasmic proteins (zonula occludens [ZOs], cingulin, etc.) and cytoskeletal proteins (actin, myosin, etc.). These transmembrane proteins polymerize on the cell membrane to form a “strand” structure, which calls TJ strand. Each TJ strand binds transversely to another one, which connects adjacent cells and forms a “zippering up” structure in the lateral to apical direction 6. Besides, cytoplasmic proteins act as scaffold proteins that connect TJ strands and cytoskeletal proteins to maintain TJ functions 9 (**Figure 1**). Cytoplasmic protein components, such as afadin and atypical protein kinase C (aPKC), also reportedly assist in the TJ structure maintenance 10, 11.

## Claudins

Claudins constitute a protein family with at least 27 subtypes 12, their molecular weight is around 20-25 kDa 13. Claudins are polymerized on the cell membrane into the backbone structure of the TJ strand. Claudins have four transmembrane fragments, two extracellular loops (the first extracellular segment [ECS1] and the second extracellular segment [ECS2]), two cytoplasmic termini (the COOH-terminus and the NH_2_-terminus). Among the claudins subtypes, ECS1 and ECS2 contain different amino acid sequences, respectively. However, ECS1 contains the claudins consensus motif, also called the “claudins signature sequence”, which performs its functions on stabilizing the structure of claudins and providing an interface for TJ^14^. About two cytoplasmic terminal fragments, the COOH-terminal fragment is almost 5 to 15 times longer than the NH_2_-terminal fragment. The number of residues of the COOH-terminal fragment is different among the claudins subtypes with residues between 21 and 63, except claudin-23 (106 residues). In claudins, the PSD-95/Dlg/ZO-1 (PDZ)-domainbinding motif at the COOH-terminal fragment can directly connects with TJ-related cytoplasmic molecules, such as ZOs and cingulin 14, 15. The PDZ domain is a motif involved in protein binding and mediates protein-protein interactions, it maintains the connection between transmembrane and cytoplasmic parts to form a complete structure and ensures TJ's function. Furthermore, it participates in various processes, such as paracellular transport and signal transduction 13.

## Occludin

Occludin is another important TJ strand component with a molecular weight around 65 kDa 16. Also, occludin has four transmembrane fragments, two extracellular loops, and two cytoplasmic termini. But they share no similarity in structure and sequence 9. In occludin, the COOH-terminal fragment has 254 residues and is about 3.88 times longer than the NH_2_-terminal fragment 17. The PDZ domain at the COOH-terminal fragment binds to ZOs to perform its functions 18.

## JAMs

JAM proteins have three main subtypes (JAM-A, JAM-B, and JAM-C), they show similar sequences and structures. JAM consists of a short NH_2_-terminal fragment, two extracellular immunoglobulin-like loops, a single transmembrane module, and a short COOH-terminal fragment in the cytoplasm with phosphorylation sites, and a COOH-terminal PDZ domain 19, 20. They molecular weight is 36-41 kDa 21. The extracellular domain functions as an adhesion structural component between cells. The phosphorylation sites are critical for JAMs to target TJs. Besides, the PDZ domain recruits TJ scaffold proteins, such as ZO-1, thus, it involves in the function of TJ through protein-protein interactions 13, 20, 22.

## ZOs

The ZOs family, including ZO-1, ZO-2, and ZO-3, are TJ scaffold proteins. They localize to the cytoplasm and the molecular weight is 220 kDa, 160 kDa, and 130 kDa, respectively 9. ZO-1, ZO-2, and ZO-3 have similar sequences. They have three PDZ domains, a Src homology 3 (SH3) domain and a guanylate kinase-like (GUK) domain. These domains are arranged in turn from NH_2_-terminus to COOH-terminus: PDZ1, PDZ2, PDZ3, SH3, and GUK 23. Among them, PDZ1 and GUK directly connect with other TJ proteins. PDZ1 binds directly to claudins COOH-terminus 9, while GUK domain binds directly to occludin NH_2_-terminus 23. Furthermore, COOH-terminal domain interacts with actin directly. But other domains do not directly connect with TJ proteins. For example, JAMs connect with the PDZ2 and PDZ3 of ZO-1 via ALL-1 fusion partner from chromosome 6 (AF-6) protein 23, 24. The ZOs are very important for maintaining the TJ structural stability. As reported, a simultaneous absence of ZO-1 and ZO-2 can cause TJ transmembrane proteins to disperse along the cytoplasm and affect TJ functions 9.

## Cingulin

Cingulin localizes in the cytoplasm. Cingulin is a parallel dimer of two subunits with a molecular weight of 140-160 kDa. Each subunit has a globular head, a coiled-coil rod, and a globular tail. Both myosin and ZO-3 can bind to the NH_2_-terminus and COOH-terminus of cingulin, while ZO-1 and ZO-2 can bind to the NH_2_-terminus 21. In general, cingulin acts as a scaffold protein that connects and stabilizes the TJ structure through ZOs family and cytoskeletal proteins. Cingulin also takes part in signal transduction, such as regulating RhoA signaling to control epithelial cell proliferation 13, 25.

## Actin

The cytoskeleton is composed of microtubules, microfilaments, and intermediate filaments. The cytoskeleton maintains cell morphology and bears external forces, it also helps maintain cell's internal structure. Microfilaments are cytoskeletal components that enable the opening and closing of TJs and are mainly composed of actin 26-28. Actin binds to the cytoplasmic scaffold proteins to form a power system that controls the opening and closing of TJ strands composed channels 29.

TJ proteins can also participate in other physiological and pathological functions independently of TJ structure. Such as, the claudins family and ZO-2 act as markers of epithelial-mesenchymal transformation (EMT) and are related to organogenesis and differentiation (EMT type 1); claudin-1, occludin, and JAM-A are related to inflammation and fibrosis (EMT type 2); the claudins family are related to cancer metastasis/invasion (EMT type 3). Claudin-1 and occludin can serve as receptors for hepatitis C virus (HCV) entry into cells 30, and ZO-1 is a biomarker of multiple myeloma to identify patients who are most likely to benefit from proteasome inhibitors 31.

## TJ functions

**Figure 2:**
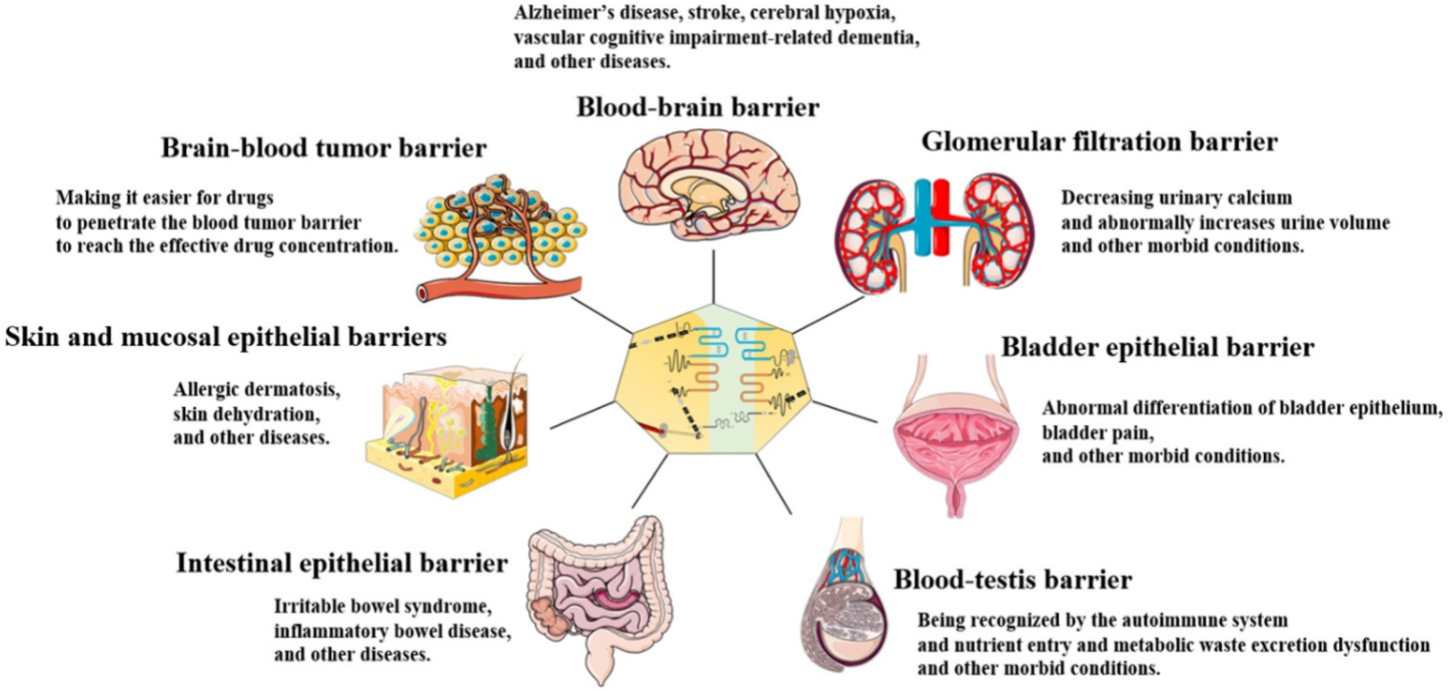
TJs are present in the skin and mucosal epithelial barriers, intestinal epithelial barrier, glomerular filtration barrier, bladder epithelial barrier, BBB, BTB, and BBTB. TJ dysfunction in these organs leads to a variety of diseases and morbid conditions.

TJs are widely found in the skin and mucosal epithelial barriers 32, intestinal epithelial barrier 33, glomerular filtration barrier^34^, bladder epithelial barrier 35, blood-brain barrier (BBB) 36, 37, brain-blood tumor barrier (BBTB) 38, and blood-testis barrier (BTB) 39, 40 (**Figure 2**). TJs can coordinate the entry and exit of molecules with different sizes and charges in an orderly manner, which plays an important role in paracellular barrier functions 41. TJs also help prevent the invasion of viruses and other microorganisms 42, maintain the polarity of epithelial cells 43, and regulate cell proliferation 44. Additionally, TJ proteins can also be expressed independently of TJ structure and play an essential role in signal transduction and regulating gene expression 31. The TJ strands of adjacent cells interact to form two types of pathways that regulate the entry and exit of molecules. The first is a pore pathway consisting of five or more TJ strands that allow ions and uncharged small molecules with a radius of approximately 4 Å or less to pass through the epithelial barrier in large numbers simultaneously. The second is the leak pathway displayed only a single strand of adjacent cells, which allows larger ions and molecules to pass through regardless of their charges, but only a small number of large ions and macromolecules can pass through at one time 41, 45. ZOs act as scaffold proteins to regulate the number of molecules passing through the two pathways 33. The claudins family regulates the charge selectivity of the two pathways, with specific residues in the ECS1 region to determine the charge selection of the pathways. The claudins family can be divided into two types: sealing claudins (claudins-1, -3, -4, -5, -8, -11, and ect), and pore-forming claudins (claudins-2, -10, -14, -16, and ect). Sealing claudins inhibit molecules passing through the TJ barrier. Furthermore, JAM-A participates in coordinating regulating TJ barrier permeability with claudins. If JAM-A alters in distribution and/or expression alone, it does not influence TJ barrier permeability. However, when JAM-A and claudins alter in distribution and/or expression together, it strengthens the influences of claudins on TJ barrier permeability 46. In contrast with sealing claudins, pore-forming claudins enable molecules passing through the TJ barrier easier 47-51. Occludin is a critical structural and functional component of TJ 18. Studies have shown that occludin constitutes a barrier function, and knockdown of occludin increases the paracellular pathway permeability 16, 52. However, some findings are opposite to this. They suggest that occludin plays a key role in maintaining the normal function of the leak pathway. The overexpression of occludin enhances leak pathway function, while the molecular flux of the leak pathway without occludin is significantly lower 53, 54. Cell polarity refers to the asymmetry of cell structure and function, and the subcellular structures are believed to be asymmetrically distributed along an axis, causing different regions inside the cell to perform different functions 55. Epithelial cells polarize along the apical-basal axis, which is crucial for the epithelial cells to function as barriers 56. In this manner, different cell-cell junctions are formed in different cell locations. TJs are located at the top of the epithelial cells, which is critical for maintaining cell polarity. For example, the absence of ZO-1 and ZO-2 induces JAM-A and claudins to be distributed widely along the apical and basolateral cell membranes, and ZO-3 and occludin are also displaced, destroying epithelial cell polarity 43. Occludin regulates the polarity of epithelial cells by controlling the Par-3/Par-6/aPKC complex 57. TJs can also regulate epithelial/endothelial cell proliferation through the Yes-associated protein 1 (YAP1) /HIPPO signaling pathway, ZO-1/zonula occludens 1‑associated nucleic acid binding protein (ZONAB) signaling pathway, and interact with integrin β1 signaling 58-62. TJ proteins can also regulate the levels of immune proteasome subunits Low-molecular mass protein 7 (LMP7) and Low-molecular mass protein 2 (LMP2) via EGFR/JAK1/signal transducer and activator of transcription 3 (STAT3) signaling independent of TJ structure 31.

TJ functions and characteristics vary between organs. TJ barrier permeability is more highly selective in the skin and bladder epithelial barriers than in the intestinal epithelial barrier and glomerular filtration barrier. This is because a strong epidermal barrier is essential for homeostasis, and the bladder must stabilize urine concentration in the normal range and prevent the loss of ion gradients 35, 63. In contrast to the skin and bladder epithelial barriers, intestinal epithelial barrier and glomerular filtration barrier drive paracellular absorption and secretion through selective TJ barrier penetration 33.

## Role of TJ in the skin and mucosal epithelial barriers

The TJ is an essential barrier structure in skin and is the main barrier in skin appendages lacking stratum corneum, such as hair follicles and sweat glands 32. The typical TJ structure is located in the granular layer of the skin 64. TJ strands form channels between adjacent keratinocytes that regulate the entry and exit of water and solutes 32. TJ deficiency leads to water and solutes losses in the skin and allows allergens entry, which in turn leads to dry skin and an overly active response to allergens. For example, atopic dermatitis is characterized by dry skin and highly active response to allergens, the two features are reported as the results of decreased claudin-1 and TJ deficiency 44, 65. Down-regulation of occludin and ZO-1 in patients with allergic rhinitis leads to TJ deficiency in nasal mucosa, and the chance of allergens crossing the TJ barrier is increased 66, 67. Defects on TJs make the skin appendages especially vulnerable to entry of viruses, such as herpes-simplex virus and infectious-molluscum virus; therefore, patients with atopic dermatitis are more likely to suffer from viral skin diseases 32. Abnormality of TJ proteins is closely related to psoriasis progression. Linkage analysis identifies nine psoriatic susceptibility loci, some of which are located in the region encoding claudins. Patients with psoriasis have been shown to lack claudin-1 expression based on the detection of skin lesions 68. *In vitro* experiments also show that the decreased expression of claudin-1 disturbs TJ functions and promotes keratinocyte proliferation 44.

## Role of TJ in the intestinal epithelial barrier

TJs perform a more complicated task in the intestinal epithelial barrier than in the skin and mucosal epithelial barriers. They support the paracellular transport of water, ions (e.g., Na^+^, Ca^2+^, Mg^2+^, and Cl^-^) and nutrients (e.g., glucose and protein), and prevent the translocation of microorganisms 33. TJs also inhibit apoptosis of intestinal epithelial cells and protect the intestinal epithelial barrier 69. Transport of Na^+^ is essential for nutrients absorption. For instance, Na^+^-glucose cotransport through TJs are vital in maintaining Na^+^ concentration inside and outside the intestinal epithelial barrier, thereby enabling nutrients recycling. In the case of TJ deficiency, the intracellular and extracellular Na^+^ concentration gradient of the intestinal epithelial barriers is insufficient to drive the absorption of transcellular Na^+^-nutrient cotransport, which then affects nutrients absorption 33. Thus, TJ deficiency can lead to intestinal epithelial absorption disorders and abnormal secretory function. TJ deficiency can cause oxalate secretion disorders, leading to hyperoxaluric acidemia, hyperoxaluria, and calcium oxalate stones. A study showed that modulation of the charge selectivity of the claudin-based pore pathway did not affect oxalate permeability, but knockdown of ZO-1 enhanced permeability to oxalate and mannitol in parallel 54. TJ deficiency is associated with inflammatory bowel disease (IBD). For example, the expressions of ZO-1 and occludin are significantly down-regulated in the intestinal epithelial barrier of patients with irritable bowel syndrome (IBS) 70, 71. Up-regulation of claudin-2, as well as down-regulation and redistribution of occludin, claudin-5, and claudin-8 lead to TJ deficiency in Crohn's disease 47. IBD increases the risk of colorectal cancer, so protecting TJ integrity is significant for preventing colorectal cancer 72. TJ defects also lead to the translocation of bacteria and bacterial products. When claudin-1 and occludin expression is down-regulated in the intestinal epithelial barrier, the barrier's permeability increases. Intestinal bacteria and their metabolites are then translocated to the liver, which can result in liver disease 42. Down-regulation of occludin expression can also cause intestinal bacteria to enter the systemic circulation, increasing cardiovascular events after myocardial infarction 73. It is worth noting that upregulation of TJ proteins can also damage the intestinal epithelial barrier, so TJ homeostasis is critical for maintaining intestinal epithelial functions.

## Role of TJ in the glomerular filtration barrier

The reabsorption by epithelial cells in the glomerular filtration barrier depends on the selection of molecular size and charge by the pathways formed by the TJ strands 34. The expression of claudins is variable in different nephron segments, which determines what will be reabsorbed in each nephron segment: claudin-2 forms cationic reabsorption pathways of proximal renal tubules; claudins-14, -16, and -19 constitute calcium transport pathways regulating the thick ascending branch of the Helen's loop; claudins-4, -7, and -8 form chloride pathways in the collecting duct; claudin-16 mediates Mg^2+^ transport protein 74, and claudins-8 and -15 mediate Na^+^-nutrient cotransport 30, 33. TJ defects also lead to reabsorption disorders. The expressions of claudins-3, -4, and -8 are down-regulated in the renal collecting duct epithelial barrier, leading to urine dilution through water reabsorption 48. Mutations in claudin-16 and claudin-19 lead to familial hypercalcemia and hypomagnesemia with renal calcinosis 74.

## Role of TJ in the bladder epithelial barrier

The selection of molecular size and charge by the pathways formed by TJ strands between bladder epithelial cells stabilizes the composition and concentration of urine in the normal range 35. Claudin-4 is a marker of bladder epithelial differentiation, and abnormal claudin-4 expression in bladder cancer is related to local invasion, lymph node metastasis, and distant metastasis 75. A study howed that claudin-4 is abnormally expressed in the bladder epithelial barrier in many children with bladder valgus 76.

## Role of TJ in the BBB

The TJ prevents harmful substances from entering the brain from the bloodstream. Down-regulation of claudin-5, ZO-1, ZO-2, and occludin causes viruses to cross the BBB 36, 37. Meanwhile, many therapeutic drugs cannot reach an effective concentration in the central nervous system due to the relative impermeability of TJs. TJ proteins disappear in the BBB from 24 hours to seven days after a stroke; only claudin-5 reappear in newly formed brain endothelial cells, whereas other components do not so. Furthermore, only claudin-5 absence does not affect TJ structure, but leads to selective opening of the BBB for molecules that smaller than 800 Da 49, 77, 78.

## Role of TJ in the BTB

TJs can protect specific antigens of germ cells from being recognized by the autoimmune system and regulate nutrient entry and metabolic waste excretion 39, 40. Unlike the BBB, TJs in the BTB can be disassembled and reassembled periodically. The BTB must be opened regularly to allow germ cells to pass. Polar proteins, for example cell division control protein 42 homolog (Cdc42), Dishevelled-1/2/3 (Dvl1/2/3), and focal adhesion kinase (FAK), alter the connection between TJ strands and cytoskeletal proteins, leading to BTB remodeling to support germ cell passage during the epithelial cycle of spermatogenesis 79-82.

## Regulatory mechanism of TJs

**Figure 3:**
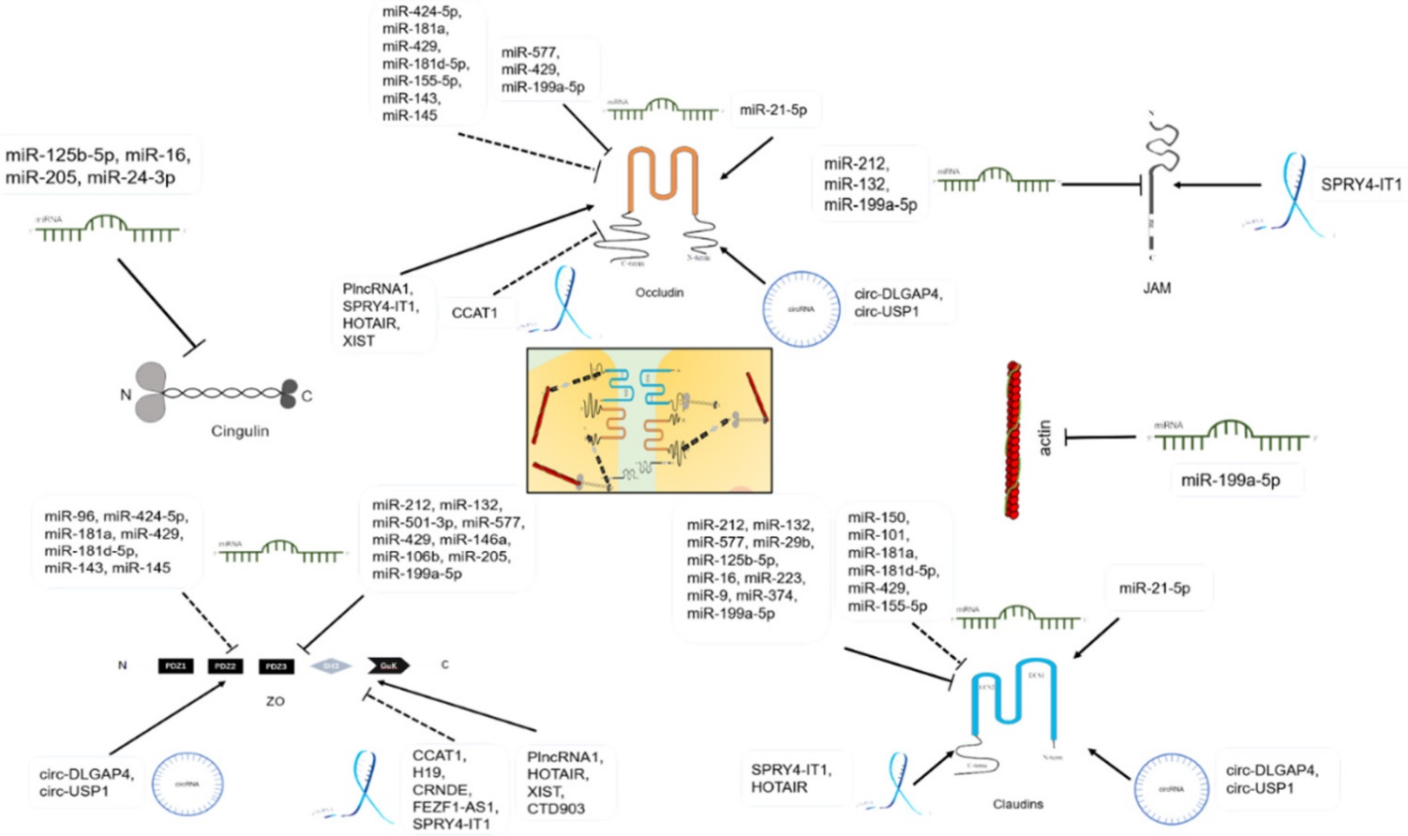
TJ proteins are regulated by miRNAs, lncRNAs, and circRNAs.

Studies currently have confirmed that a variety of signaling pathways can affect TJ by regulating TJ protein expression: Ras-mitogen-activated protein kinase (MAPK), epidermal growth factor receptor (EGFR)-Src family kinases (SFK), Toll-like receptor 2 (TLR2)-protein kinase C (PKC), Wnt- β-catenin, Nrf2, NF-κB, STAT5b, and other signaling pathways can regulate the expression of ZO-1^83^-^93^; mitogen-activated protein kinase (MAPK) kinase (MEK)-extracellular signal-regulated kinase (ERK), Nrf2, NF-κB, mediterranean fever gene (MEFV), and other signaling pathways can regulate occludin expression 72, 89-94; the Nrf2 pathway regulates claudin-4 expression 90, 91; the Wnt-β-catenin and c-myc pathways regulate claudin-5 expression 86-88, 95; the STAT5b pathway regulates the expressions of ZO-2 and ZO-3 96; the Siah-Pard3A pathway regulates JAM-A expression 97; and the liver kinase B1 (LKB1)/adenosine monophosphate-activated protein kinase (AMPK) pathway regulates cingulin expression 98. Epigenetic regulation, such as DNA methylation and histone modifications, also plays an important role in maintaining TJ function. DNA methylation in the promoter regions of TJ-related genes can inhibit their expressions. For instance, DNA methylation inhibits claudin-11 expression by interfering with transcription activator GATA1 binding to its DNA promoter 99. Histone modification in the promoter region of the TJ gene can also affect TJ gene transcription: both histone 3 lysine 9 trimethylation (H3K9me3) and histone 3 lysine 27 trimethylation (H3K27me3) in the ZO-1 DNA promoter inhibit ZO-1 transcriptional expression 100-102, while histone 3 lysine 4 trimethylation (H3K4me3) of the ZO-1 DNA promoter activates ZO-1 DNA transcription 101; besides, H3K27me3 and histone 4 lysine 20 trimethylation (H4K20me3) of claudin-3 and claudin-4 DNA promoters inhibit their transcription 103; and H3K27me3 of the occludin DNA promoter inhibits occludin transcriptional expression 101, 102. Non-coding RNAs, including micro-RNAs (miRNAs), long noncoding RNAs (lncRNAs), and circular RNAs (circRNAs) are also vital aspects of epigenetic regulation, they play an important role in the physiological and pathological regulation of various genes. In recent years, it has been reported that non-coding RNAs are also involved in TJ function regulation (**Figure 3**).

## Non-coding RNAs

MiRNAs are small single-stranded RNAs with lengths of 21-23 nucleotides that can bind to the 3' UTR of mRNAs and result in mRNAs silencing or degradation 104, 105. For example, miR-29 can participate in the pathogenesis of recessive dystrophic epidermolysis bullae by specifically silencing COL7A1 mRNA 106. LncRNAs are long-stranded non-coding RNAs with lengths of 200-100,000 nucleotides that participate in the regulation of a variety of physiological and pathological processes 107, 108. The regulation mechanisms of lncRNAs are highly complex, but they mainly work in the following ways 109-123: (1) regulating gene transcription by affecting DNA promoter regions; (2) inhibiting RNA polymerase II or regulating chromatin remodeling and histone modification to affect downstream gene expression; (3) forming a complementary double strand with gene transcripts to interfere with mRNA splicing; (4) forming complementary double strands with the transcript of genes to produce endogenous siRNAs dependent on Dicer enzyme activity; (5) binding to a specific protein to regulate its activity; (6) binding to a specific protein to change its cellular location; (7) acting as a structural component, forming a nucleic acid-protein complex with protein; (8) a precursor of small RNAs such as miRNAs and piRNAs; (9) sponge-like absorbing of miRNAs to indirectly regulate gene expression that targeted by miRNA. CircRNA is a single-stranded RNA with a closed loop structure that is more stable than linear RNA. CircRNA exerts its biological functions mainly through sponging miRNAs, binding proteins, regulating gene transcription, or being translated into short polypeptide as templates 124-129. For example, circ_0015756 can sponge miR-1250-3p to regulate the proliferation and invasiveness of hepatoblastoma 130. Circ-human antigen R (HuR) has been shown to be down-regulated in gastric cancer and bound to the CCHC zinc-finger nucleic acid-binding protein (CNBP), which promotes HuR mRNA expression by interacting with the HuR DNA promoter. However, once bound to CNBP, circ-HuR competitively inhibits CNBP binding with the HuR DNA promoter. Thereby, it down-regulates HuR to inhibit the progression of gastric cancer 127. Previous studies have shown that DNA methyltransferase 1 (DNMT1) contributes to the DNA methylation in CD4^+^ T cells, which leads to the production of autoantibodies. Down-regulation of circ_0012919 expression increases methyltransferase DNMT1 expression, which contributes to DNA methylation of CD11a and CD70 in CD4^+^T cells. Therefore, circ_0012919 can be regarded as a biomarker of systemic lupus erythematosus 131. There are also small nuclear RNAs (snRNAs), transfer RNAs (tRNAs), small interfering RNAs (siRNAs), and other undiscovered non-coding RNAs in cells that regulate a variety of cellular processes.

## MiRNAs and TJs

The gene locations and sequences of miRNAs are highly conserved and are found throughout animals, plants, bacteria, fungi, and viruses 105, 132. It has been reported that miRNAs participate in different kinds of physiological functions such as post-transcriptional gene-expression 133, cardiovascular biomarkers 134, angiogenesis, and inflammatory response 135. Disordered miRNAs expressions also participate in the regulation of pathological processes such as tumor pathogenesis 136, drug resistance 137. It has been found that miRNAs can regulate physiological and pathological processes by regulating TJs in the BBB, BBTB, intestinal epithelial barrier, skin epithelial barrier, glomerular filtration barrier, bladder epithelial barrier, and EMT via regulating TJ proteins (**Table 1**). The BBB is the barrier between plasma and brain cells formed by the cerebral capillary wall and glial cells, as well as the barrier between plasma and cerebrospinal fluid formed by the choroid plexus. The BBB protects brain tissue from harmful substances circulating in the blood and maintains the stability of the environment in the brain tissue 138. The BBTB is the barrier between brain tumor tissue and plasma formed by the capillary wall in brain tumor tissue 138. The intestinal epithelial barrier prevents bacteria and toxins from passing through the intestinal mucosa into other tissues, organs, and blood circulation in the human body. It can also promote the absorption of nutrients, such as proteins, into the bloodstream 139. The skin and mucosal epithelial barriers are composed of the physical barrier and the chemical barrier. They both protect the internal organs/tissues from external damage, irritation, and sunlight entry, and they function in skin moisturization and immunomodulation. TJs are mainly important in the physical barrier of the skin and mucosal epithelial barriers 140-142. Glomerular filtration barrier is the structure in which blood in the glomerular capillaries is filtered into Bowman space 143, 144. The bladder epithelial barrier is the mucous membrane that lines the inner surface of the bladder 145. EMT is the transformation from epithelial cells to mesenchymal cells, which gives cells the ability to migrate and invade other tissues. The EMT has a key role in tissue healing, organ fibrosis, carcinogenesis, and other processes 146.

## MiRNAs and TJs in the BBB

The BBB damage will lead to stroke, vascular cognitive impairment-related dementia, Alzheimer's disease, cerebral hypoxia, and other conditions 147-152. The most important TJ proteins in the BBB are claudin-5, ZO-1, and occludin. MiR-150 directly binds to angiopoietin receptor Tie-2 mRNA, resulting in the down-regulation of Tie-2 protein, which leads to the inhibition of claudin-5 protein, and an increase in the BBB permeability after ischemic stroke 149. Hypoxia induces high miR-212 and miR-132 in the central nervous system. Both miR-212 and miR-132 can bind to the 3' UTR of claudin-1, ZO-1, and JAM-C mRNAs and inhibit their expressions, thereby damage the integrity of the BBB 150. MiR-501-3p binds to the 3' UTR of ZO-1 mRNA to inhibit ZO-1 protein expression; this leads to damage of the BBB and promotes development of vascular cognitive impairment-related dementia 151. Current studies have shown that Alzheimer's disease is closely related to an increase in monocyte migration across the BBB. High level of granulocyte-macrophage colony-stimulating factor (GM-CSF) in brain parenchyma promotes monocyte migration across the BBB. GM-CSF up-regulates the miR-96 level of human brain microvascular endothelial cells (HBMEC) via the phosphatidylinositol 3-kinase (PI3K)/serine-threonine protein kinase (AKT) signal pathway. MiR-96 targets the mRNA of the erythrocyte transformation specificity (ETS) transcription factor, ETS-related gene (ERG), to inhibit ERG protein expression. The interaction of ERG protein with the ZO-1 DNA promoter region enables ZO-1 mRNA expression. Therefore, miR-96 indirectly suppresses ZO-1 mRNA expression, which leads to an increase in monocyte migration across the BBB 148. MiR-424-5p binds to the 3' UTR of endophilin-1 mRNA and up-regulates endophilin-1 mRNA. Endophilin-1 overexpression leads to inhibition of the EGFR-ERK and EGFR-Jun-N-terminal kinase (JNK) signaling pathways, then down-regulates ZO-1 and occludin protein in vascular endothelial cells. This increases the BBB permeability and induces development of Alzheimer's disease 152. It is worth noted that the combination of miR-424-5p with the endophilin-1 mRNA 3' UTR does not decrease endophilin-1 mRNA. This may be due to a competitive interaction between miR-424-5p and multiple miRNAs, which reduce the inhibition of other miRNAs on endophilin-1 expression and indirectly increase endophilin-1 153. MiR-101 binds to the 3' UTR of vascular endothelial cadherin (VE-cadherin) mRNA to down-regulate it. VE-cadherin has been reported to promote claudin-5 expression. Therefore, miR-101 indirectly down-regulates claudin-5, resulting in the increase of BBB permeability 154.

## MiRNAs and TJs in the BBTB

Research has focused on drugs or treatments that increase the permeability of the BBTB to enable an effective drug concentration to penetrate the BBTB and treat brain tumors. Studies have found that miRNAs can increase the BBTB permeability by regulating TJs to achieve increased drug efficacy, which is significant for clinical advancement. The expression of miR-181a is increased in glioma, and miR-181a can inhibit Kruppel-like factor 6 (KLF6) mRNA expression by directly binding to the 3' UTR of KLF6 mRNA; KLF6 can promote the transcriptional expressions of ZO-1, occludin, and claudin-5 mRNAs. Thus, miR-181a indirectly down-regulates ZO-1, occludin, and claudin-5, which further increases the BBTB permeability. MiR-181a may be a key miRNA for opening the BBTB and is a potential target to increase the therapeutic success rate for glioma 38. MiR-577 directly binds to occludin, ZO-1, and claudin-1 mRNA 3' UTRs, down-regulating they and increasing the BBTB permeability to improve the chemosensitivity of malignant glioma 155. MiR-429 expression in human glioma microvascular endothelial cells (GECs) is much lower than that in normal microvascular endothelial cells in human brain. MiR-429 regulates TJ proteins in GECs in two ways. First, miR-429 directly binds to ZO-1 and occludin mRNA 3' UTRs to inhibit the post-transcriptional processes. Second, miR-429 inhibits p70 S6 kinase-S6 (p70S6K-S6) expression by binding to the mRNA 3' UTR. The activation of the p70S6K-S6 signaling pathway can up-regulate ZO-1, occludin, and claudin-5. Therefore, miR-429 can also indirectly down-regulate ZO-1, occludin, and claudin-5 by inhibiting the p70S6K-S6 signaling pathway 156. The expressions of ZO-1, occludin, and claudin-5 are also promoted by binding of Sex-determining region Y-box protein 5 (SOX5), whose expressions are inhibited by miR-181d-5p. Thus, miR-181d-5p could suppress SOX5 expression to reduce ZO-1, occludin, and claudin-5 expressions, thereby enhancing the BBTB permeability in glioma 157.

## MiRNAs and TJs in the intestinal epithelial barrier

The destruction of the intestinal epithelial barrier leads to IBS, IBD, and other conditions. The function of the intestinal epithelial barrier is complex, and the TJ proteins, which predominate in the barrier structure, have intricate functions 158-161. It has been found that miR-29b combines with the 3' UTR of claudin-1 mRNA to inhibit claudin-1 expression, resulting in intestinal epithelial barrier dysfunction 158. Intestinal symbiotic floras induce the expression of miR-21-5p in intestinal epithelial cells. MiR-21-5p binds to phosphatase and tensin homolog (PTEN), programmed cell death factor 4 (PDCD4), sprouty 1 (SPRY1), and sprouty 2 (SPRY2) mRNAs to inhibit their expressions. These target genes are negative regulators of the phosphatidylinositol 3-kinase (PI3K)-Akt, JNK-activator protein 1 (AP-1), and ERK pathways, respectively. Therefore, miR-21-5p activates these pathways to up-regulate ADP ribosylation factor 4 (ARF4), promoting the expressions of claudin-4 and occludin and protecting the intestinal epithelial integrity 159. MiR-125b-5p and miR-16 bind to cingulin and claudin-2 mRNAs and inhibit their expressions, respectively. In IBS, however, miR-125b-5p and miR-16 are down-regulated, which increase cingulin and claudin-2 in the intestinal epithelial barrier and leads to intestinal epithelial barrier dysfunction 160. MiR-223 directly binds to the 3' UTR of claudin-8 mRNA and down-regulate it, increasing the permeability of intestinal epithelial barrier and inducing IBD occurrence 161.

## MiRNAs and TJs in the skin and mucosal epithelial barriers

TJ destruction caused by abnormal miRNA expression induces allergic dermatosis, skin dehydration, and other diseases 162, 163. For example, miR-155-5p binds to the protein kinase inhibitor α (PKI α) mRNA 3'UTR to inhibit PKI α expression. PKI α can promote claudin-1 and occludin expressions, while miR-155-5p can increase skin barrier permeability and induce atopic dermatitis by indirectly down-regulating claudin-1 and occluding 162. Both miR-146a and miR-106b bind to the ZO-1 and ZO-2 mRNA 3'UTRs to inhibit their expressions, increasing skin barrier permeability, and increasing trans-epidermal water loss (TEWL) 163.

## MiRNAs and TJs in the glomerular filtration barrier

The epithelium of the glomerular filtration barrier reabsorbs water, electrolytes, and nutrients in urine mainly through TJs. TJ destruction decreases urinary calcium abnormally increases urine volume and causes other morbid conditions 50, 164. Both miR-9 and miR-374 recognize complementary binding sites in the claudin-14 mRNA 3' UTR. The miR-9/miR-374-claudin-14 pathway is directly regulated by Ca^2+^ sensor receptor (CaSR) and the pathway also constitutes an important part of the renal CaSR signal cascade system. CaSR activation decreases miR-9 and miR-374 expressions, then increases claudin-14, which increases calcium reabsorption and decreases urinary calcium by increasing TJ barrier permeability 50, 164.

## MiRNAs and TJs in the bladder epithelial barrier

Damage to the TJs between adjacent bladder epithelial cells leads to abnormal differentiation of the bladder epithelial barrier, bladder pain, and other morbid conditions 165-167. It was reported that miR-199a-5p increases bladder epithelial permeability in bladder pain syndrome by targeting the 3' UTRs of ZO-1, JAM-A, occludin, and actin mRNA 165. Similarly, miR-199a-5p up-regulation in bladder pain syndrome leads to occludin, claudin-1, and JAM-A down-regulation, which is not conducive to the establishment of a tight bladder epithelial barrier and results in bladder chronic pain 167. MiR-205 directly binds to ZO-1 and cingulin mRNAs to inhibit their expressions and participates in urothelial cell differentiation regulation 166.

## MiRNAs regulate EMT via TJs

EMT leads to TJ destruction and the loss of cell adhesion and polarity. Damage to TJ structural integrity can also promote EMT 168, 169. MiR-143 and miR-145 inhibit cAMP-response element binding protein 1 (CREB1) expression, which is the transcriptional activator of ZO-1, ZO-3, and occludin. So miR-143 and miR-145 down-regulate these TJ proteins, promoting breast cancer invasion and metastasis induced by EMT 168. MiR-24-3p inhibits cingulin mRNA translation, which leads to invasion and metastasis of malignant mesothelioma induced by EMT 169.

## LncRNAs and TJs

It has been reported that lncRNAs regulate the physiological functions of cell directional differentiation, biological growth and evolution 170. Disordered lncRNAs expressions also participate in the regulation of pathological processes such as tumor progression, cardiovascular and cerebrovascular diseases, neurological diseases, autoimmune diseases, and congenital malformations 104, 105, 107, 108. In recent years, it has been found that lncRNAs can regulate physiological and pathological processes by regulating TJs in the intestinal epithelial barrier, BBTB, and EMT (**Table 2**).

## LncRNAs and TJs in the intestinal epithelial barrier

Colon cancer-associated transcript-1 (CCAT1) performs an important role in tumorigenesis and progression. CCAT1 can also induce IBD by regulating the intestinal epithelial barrier. For example, inflammation-related genes are significantly enriched in colorectal cancer patients with high CCAT1 expression. Myosin light chain kinase (MLCK) can induce the disordered distribution of occludin and ZO-1 and can transform the intestine from a smooth arc shape into many irregular undulations. CCAT1 can adsorb miR-185-3p to promote MLCK expression, leading to disordered distribution of occludin and ZO-1 and destruction of the barrier function, which promotes IBD pathogenesis 171. H19 expression increases in patients with ulcerative colitis. H19, as a precursor of miR-675-5p, inhibits ZO-1 mRNA expression and induces intestinal epithelial barrier function destruction in patients with ulcerative colitis 172, 173. PlncRNA1 sponges miR-34c to remove the targeted inhibition of miR-34c on myc-associated zinc-finger protein (MAZ). MAZ promotes ZO-1 and occludin expressions by binding to ZO-1 and occludin promoter regions. Therefore, PlncRNA1 can up-regulate occludin and ZO-1 through the “miR-34c-MAZ” regulatory axis and enhance the intestinal epithelial barrier function 174. SPRY4 intronic transcript 1 (SPRY4-IT1) directly interacts with occludin, claudin-1, claudin-3, and JAM-A mRNAs to promote their translation by binding to the RNA-binding protein HuR, and then enhances intestinal epithelial barrier function 175.

## LncRNAs and TJs in the BBTB

HOX transcript antisense intergenic RNA (HOTAIR) is up-regulated in gliomas, and HOTAIR combines with the promoter regions of occludin, ZO-1, and claudin-5 to promote their expressions, which decreases BBTB permeability 176. The adsorption of miR-137 by X inactivate-specific transcript (XIST) can antagonize the targeted inhibition of miR-137 on ZO-2. So XIST promotes ZO-2 expression. In addition, XIST can also promote forkhead box C1 (FOXC1) expression by adsorbing miR-137. FOXC1 is a gene transcription factor of ZO-1 and occludin. Therefore, XIST can up-regulate ZO-1, ZO-2, and occludin from multiple angles, which decreases BBTB permeability 177.

## LncRNAs regulate tumor EMT through TJs

LncRNAs can participate in tumor EMT regulation by regulating TJ proteins. For example, colorectal neoplasia differentially expressed (CRNDE) is highly expressed in osteosarcoma and hepatocellular carcinoma cells. CRNDE activates the Wnt/β-catenin signaling pathway and down-regulates ZO-1, inducing EMT to promote osteosarcoma and hepatocellular carcinoma cell metastasis 178, 179. FEZF1 antisense RNA 1 (FEZF1- AS1) expression is significantly up-regulated in non-small cell lung cancer (NSCLC) tissues. FEZF1-AS1 also down-regulates ZO-1, inducing EMT by activating the Wnt/β-catenin signal pathway 180. CTD903 expression is significantly up-regulated in colorectal cancer tissue. CTD903 inhibits the Wnt/β-catenin signaling pathway to up-regulate ZO-1, inhibiting EMT in colorectal cancer 181. SPRY4-IT1 down-regulates ZO-1 by promoting Snail transcription, expression, and nuclear localization, inducing EMT and metastasis of esophageal squamous cell carcinoma 182.

## CircRNAs and TJs

There have been found a few studies of TJ regulation by circRNAs (**Table 3**). However, studies have introduced the role of circRNAs in the BBB and BBTB. For example, circ-DLGAP4 is highly expressed in the normal central nervous system, but significantly decreased after stroke, resulting in down-regulation of TJ protein and BBB destruction. The combination of miR-143 and HECT domain E3 ubiquitin protein ligase 1 (HECTD1) mRNA 3' UTR inhibits HECTD1 expression. HECTD1 can promote the expressions of occludin, ZO-1, and claudin-5. Circ-DLGAP4 can promote HECTD1 expression by sponging miR-143 and enhance BBB function 183. As a miR-194-5p sponge, circ-ubiquitin specific peptidase 1 (USP1) relieves the inhibitory effect of miR-194-5p on occludin, ZO-1, and claudin-5 mRNAs and decreases the BBTB permeability 184. CircRNAs are stable and have potential as molecular markers and drug targets, and their relationships with TJs deserve more extensive and in-depth researches.

## Conclusion

A TJ is a “zippering up” junction structure. TJs maintain the relative stability of intracellular substances and functions by closing or opening paracellular pathways, coordinating the entry and exit of molecules of different sizes and charges, and regulating the permeability of paracellular barrier. TJs also prevent microbial invasion, maintain epithelial/endothelial cell polarity, and regulate cell proliferation. TJs are widely present in the skin and mucosal epithelial barriers, intestinal epithelial barrier, glomerular filtration barrier, bladder epithelial barrier, BBB, BBTB, and BTB. Because of TJ's critical role in organs and tissues, it is not surprising that diseases, such as IBD, gliomas, allergic dermatosis, and skin dehydration, are associated with disruption of TJs. A variety of non-coding RNAs, such as miRNAs, lncRNAs, and circRNAs, can directly or indirectly regulate TJ proteins, affecting the function of various organs or leading to diseases.

## Prospect

Non-coding RNAs are important parts of epigenetic regulation. Current studies have confirmed that many miRNAs can affect the TJs by directly regulating TJ proteins or indirectly regulating upstream signal pathways or transcription factors of TJ proteins, thereby affecting the skin and mucosal epithelial barriers, intestinal epithelial barrier, BBB, EMT and others. However, the research on the roles of lncRNAs, circRNAs, and other non-coding RNAs in TJs is still in its infancy. Because of the diversity of mechanisms and functions of lncRNAs and circRNAs, more in-depth studies are needed to reveal their roles and specific mechanisms. The role of snRNAs, tRNAs and other non-coding RNAs in TJs also needs to be confirmed by follow-up research. Because the disorder of TJs can lead to a variety of diseases, such as skin barrier dysfunction, which will lead to the progression of atopic dermatitis, psoriasis, ichthyosis, melanoma and so on. TJ proteins and non-coding RNAs that regulate TJs are expected to become new therapeutic targets. Some drugs targeted at TJs may also promote the drugs to enter the lesion and increase the sensitivity of the drugs.

## Figures and Tables

**Table 1:** Function and mechanism of miRNAs in TJs

Name	Level	Function	Mechanism	Position	Reference
miR-150	-	Increasing BBB permeability	Down-regulating claudin-5 by target inhibiting Tie-2	BBB	149
miR-212 and miR-132	Increased in hypoxic brain tissue	Destroying the integrity of BBB	Target inhibiting claudin-1, ZO-1, and JAM-C	BBB	150
miR-501-3p	-	Destructing the integrity of BBB	Target inhibiting ZO-1	BBB	151
miR-96	Increased in HBMEC of Alzheimer's disease	Increasing BBB permeability	Down-regulating ZO-1 by target inhibiting ERG	BBB	148
miR-424-5p	-	Increasing BBB permeability	Down-regulating ZO-1 and occludin by target promoting Endophilin-1	BBB	152
miR-101	-	Increasing BBB permeability	Down-regulating claudin-5 by target inhibiting VE-cadherin	BBB	154
miR-181a	Increased in Glioma	Increasing BBTB permeability	Down-regulating ZO-1, occludin, and claudin-5 by target inhibiting KLF6	BBTB	38
miR-577	-	Increasing BBTB permeability	Target inhibiting claudin-1, ZO-1, and occludin	BBTB	155
miR-429	Decreased in Glioma	Increasing BBTB permeability	Target inhibiting ZO-1 and occludin. Also, down-regulating ZO-1, occludin, and claudin-5 by target inhibiting p70S6K-S6	BBTB	156
miR-181d-5p	-	Increasing BBTB permeability	Down-regulating ZO-1, occludin, and claudin-5 by target inhibiting SOX5	BBTB	157
miR-29b	-	Destroying the integrity of intestinal epithelial barrier	Target inhibiting claudin-1	Intestinal epithelial barrier	158
miR-21-5p	Decreased in dysfunctional intestinal epithelial barrier	Protecting the integrity of intestinal epithelial barrier	Up-regulating occludin and claudin-4 by target inhibiting PTEN, PDCD4, SPRY1, and SPRY2	Intestinal epithelial barrier	159
miR-125b-5p and miR-16	Decreased in irritable bowel syndrome	Protecting the integrity of intestinal epithelial barrier	Target inhibiting cingulin and claudin-2	Intestinal epithelial barrier	160
miR-223	-	Destroying the integrity of intestinal epithelial barrier	Target inhibiting claudin-8	Intestinal epithelial barrier	161
miR-155-5p	-	Increasing skin barrier permeability	Down-regulating occludin and claudin-1 by target inhibiting PKIα	Skin epithelial barrier	162
miR-146a and miR-106b	-	Increasing skin barrier permeability and TEWL	Target inhibiting ZO-1 and ZO-2	Skin epithelial barrier	163
miR-9 and miR-374	-	Increasing glomerular filtration barrier permeability leading to an increase in calcium reabsorption and a decrease in urinary calcium	Target inhibiting claudin-14	Glomerular filtration barrier	50, 164
miR-205	-	Regulating urinary tract epithelial cell differentiation	Target inhibiting cingulin and ZO-1	Urinary tract epithelium	166
miR-199a-5p	Increased in the bladder pain syndrome	Increasing bladder epithelial permeability	Target inhibiting ZO-1, JAM-A, occludin, and actin	Bladder epithelium	165
miR-199a-5p	Increased in the bladder pain syndrome	Increasing bladder epithelial permeability	Target inhibiting claudin-1, JAM-A, and occludin	Bladder epithelium	167
miR-143 and miR-145	-	Promoting EMT	Down-regulating occluding, ZO-1, and ZO-3 by target inhibiting CREB1	Breast cancer cells	168
miR-24-3p	-	Promoting EMT	Target inhibiting cingulin	Malignant mesothelioma	169

Abbreviation: BBB: blood-brain barrier; BBTB: brain blood-tumor barrier; CREB1: cAMP-response element binding protein 1; EMT: epithelial to mesenchymal transition; ERG: ETS-Related Gene; JAM-A: junctional adhesion molecule A; JAM-C: junctional adhesion molecule C; KLF6: Kruppel-like factor 6; p70S6K-S6: p70 S6 kinase-S6; PDCD4: programmed cell death factor 4; PKIα: protein kinase inhibitor α; PTEN: phosphatase and tensin homolog; SOX5: sex-determining region Y-box protein 5; SPRY1: sprouty 1; SPRY2: sprouty 2; TEWL: trans-epidermal water loss; VE-cadherin: vascular endothelial cell cadherin; ZO-1: zonula occludens-1; ZO-2: zonula occludens-2; ZO-3: zonula occludens-3.

**Table 2:** Function and mechanism of lncRNAs in TJs

Name	Level	Function	Mechanism	Position	Reference
CCAT1	-	Destroying intestinal epithelial barrier function and promoting the pathogenesis of inflammatory bowel disease.	Disturbing ZO-1 and occludin expressions by sponging miR-185-3p	Intestinal epithelial barrier	171
H19	Increased in ulcerative colitis	Destroying the intestinal epithelial barrier	Down-regulating ZO-1 by releasing miR-675-5p	Intestinal epithelial barrier	172,173
PlncRNA1	-	Protecting intestinal epithelial barrier function	Up-regulating ZO-1 and occludin by sponging miR-34c	Intestinal epithelial barrier	174
SPRY4-IT1	-	Protecting intestinal epithelial barrier function	Target promoting occludin, claudin-1, claudin-3, and JAM-A translation	Intestinal epithelial barrier	175
HOTAIR	Increased in glioma	Decreasing BBTB permeability	Target up-regulating occludin, ZO-1, and claudin-5	BBTB	176
XIST	-	Decreasing BBTB permeability	Up-regulating ZO-1, ZO-2, and occludin by sponging miR-137	BBTB	177
CRNDE	Increased in osteosarcoma and hepatoma cells	Promoting EMT	Down-regulating ZO-1 by target activating Wnt/β-catenin signal pathway	Osteosarcoma and hepatoma	178,179
FEZF1-AS1	Increased in NSCLC	Promoting EMT	Down-regulating ZO-1 by target activating Wnt/β-catenin signal pathway	NSCLC	180
CTD903	Increased in colorectal cancer	Inhibiting EMT	Up-regulating ZO-1 by target inhibiting Wnt/β-catenin signal pathway	Colorectal cancer	181
SPRY4-IT1	-	Promoting EMT	Down-regulating ZO-1 by target activating Snail transcription, expression, and nuclear localization	Esophageal squamous cell carcinoma	182

Abbreviation: BBTB: brain blood-tumor barrier; CCAT1: colon cancer-associated transcript-1; CRNDE: colorectal neoplasia differentially expressed; EMT: epithelial to mesenchymal transition; FEZF1-AS1: FEZF1 antisense RNA 1; HOTAIR: HOX transcript antisense intergenic RNA; JAM-A: junctional adhesion molecule A; NSCLC: non-small cell lung cancer; SPRY4-IT1: SPRY4 intronic transcript 1; XIST: X inactivate-specific transcript; ZO-1: zonula occludens-1; ZO-2: zonula occludens-2.

**Table 3:** Function and mechanism of circRNAs in TJs

Name	Level	Function	Mechanism	Position	Reference
circ-DLGAP4	Decreased in ischemic brain tissue	Protecting BBB	Up-regulating ZO-1, claudin-5 and occludin by sponging miR-143	BBB	183
circ-USP1	-	Decreasing BBTB permeability	Up-regulating ZO-1, claudin-5, and occludin by sponging miR-194-5p	BBTB	184

Abbreviation: BBB: blood-brain barrier; BBTB: brain blood-tumor barrier; USP1: ubiquitin specific peptidase 1; ZO-1: zonula occludens-1.

## Abbreviations

AF-6: ALL-1 fusion partner from chromosome 6
AKT: serine-threonine protein kinase
AMPK: adenosine monophosphate-activated protein kinase
AP-1: activator protein 1
aPKC: atypical protein kinase C
ARF4: ADP ribosylation factor 4
BBB: blood-brain barrier
BBTB: brain blood-tumor barrier
CaSR: Ca-sensing receptor
CCAT1: colon cancer-associated transcript-1
Cdc42: cell division control protein 42 homolog
CNBP: CCHC Zinc finger nucleic acid-binding protein
COL7A1: collagen, type VII, alpha 1
CREB1: cAMP-response element binding protein 1
CRNDE: colorectal neoplasia differentially expressed
DNMT1: DNA methyltransferase 1
Dvl1/2/3: Dishevelled-1/2/3
ECS1: the first extracellular segment
ECS2: the second extracellular segment
EGFR: epidermal growth factor receptor
EMT: epithelial to mesenchymal transition
ERG: ETS-Related Gene
ERK: extracellular signal-regulated kinase
ETS: erythrocyte transformation specificity
FAK: focal adhesion kinase
FEZF1-AS1: FEZF1 antisense RNA 1
FOXC1: forkhead box C1
GECs: glioma microvascular endothelial cells
GM-CSF: granulocyte-macrophage colony-stimulating factor
GUK: guanylate kinase-like
HCV: hepatitis C virus
HBMEC: human brain microvascular endothelial cells
HECTD1: HECT domain E3 ubiquitin protein ligase 1
HOTAIR: HOX transcript antisense intergenic RNA
H3K4me3: histone 3 lysine 4 trimethylation
H3K9me3: histone 3 lysine 9 trimethylation
H3K20me3: histone 3 lysine 20 trimethylation
H3K27me3: histone 3 lysine 27 trimethylation
HuR: human antigen R
IBD: inflammatory bowel disease
IBS: irritable bowel syndrome
JAM-A: junctional adhesion molecule A
JAM-C: junctional adhesion molecule C
JNK: Jun-N-terminal kinase
KLF6: Kruppel-like factor 6
LKB1: liver kinase B1
LMP2: Low-molecular mass protein 2
LMP7: Low-molecular mass protein 7
MAPK: mitogen-activated protein kinase
MAZ: myc-associated zinc-finger protein
MEK: mitogen-activated protein kinase (MAPK) kinase
MLCK: myosin light chain kinase
NF-κB: nuclear factor-kappa B
NSCLC: non-small cell lung cancer
p70S6K-S6: p70 S6 kinase-S6
PDCD4: programmed cell death factor 4
PDZ: PSD-95/Dlg/ZO-1
PI3K: phosphatidylinositol 3-kinase
PKIα: protein kinase inhibitor α
PKC: protein kinase C
PTEN: phosphatase and tensin homolog
SH3: Src homology 3
snRNAs: small nuclear RNAs
siRNAs: small interfering RNA
SOX5: sex-determining region Y-box protein 5
SFK: Src family kinases
SPRY1: sprouty 1
SPRY2: sprouty 2
SPRY4-IT1: SPRY4 intronic transcript 1
STAT5b: signal transducer and activator of transcription 5b
TEWL: trans-epidermal water loss
TJ: tight junction
TLR2: Toll-like receptor 2
tRNAs: transfer RNAs
USP1: ubiquitin specific peptidase 1
VE-cadherin: vascular endothelial cell cadherin
XIST: X inactivate-specific transcript
YAP1: Yes-associated protein 1
ZO-1: zonula occludens-1
ZO-2: zonula occludens-2
ZO-3: zonula occludens-3
ZONAB: zonula occludens 1‑associated nucleic acid binding protein
